# Beta-blocker choice and exchangeability in patients with heart failure and chronic obstructive pulmonary disease: an Italian register-based cohort study

**DOI:** 10.1038/s41598-019-47967-y

**Published:** 2019-08-07

**Authors:** Maurizio Sessa, Annamaria Mascolo, Daniel Bech Rasmussen, Kristian Kragholm, Magnus Thorsten Jensen, Liberata Sportiello, Concetta Rafaniello, Giuseppe Michele Tari, Claudia Pagliaro, Morten Andersen, Francesco Rossi, Annalisa Capuano

**Affiliations:** 1 Department of Drug Design and Pharmacology, University of Copenhagen, Jagtvej 160, 2100 København Ø, Denmark; 2 Campania Pharmacovigilance and Pharmacoepidemiology Regional Centre, Department of Experimental Medicine, University of Campania “L. Vanvitelli”, Via Santa Maria di Costantinopoli 16, 80138 Naples, Italy; 3 Respiratory Research Unit Zealand, Department of Respiratory Medicine, Naestved Hospital, Ringstedgade 61, 4700 Næstved, Denmark; 4 Department of Cardiology, Herlev and Gentofte University Hospital, Gentofte Hospitalsvej 1, 2900 Hellerup, Denmark; 5 Unit of Epidemiology and Biostatistics, Aalborg University Hospital, Hobrovej 18-22, 9100 Aalborg, Denmark; 6 Heart Centre, Copenhagen University Hospital Rigshospitalet, Blegdamsvej 9, 2100 København, Denmark; 7 Caserta Local Health Unit, Via Unità Italiana 28, 81100 Caserta, Italy

**Keywords:** Interventional cardiology, Epidemiology

## Abstract

Clinical guidelines suggest that for patients with heart failure and concurrent chronic obstructive pulmonary disease (COPD), metoprolol/bisoprolol/nebivolol should be preferred over carvedilol. However, studies suggest a high proportion of carvedilol usage that remains unexplained. Therefore, we aimed to investigate the predictors of carvedilol choice in patients with heart failure and COPD that were naïve to carvedilol or metoprolol/bisoprolol/nebivolol. Caserta Local Health Unit databases (Italy) were used as data sources. Age, sex, chronic/acute comorbidities, and co-medications were included in a logistic regression model to assess predictors of carvedilol choice. Chronic comorbidities include those defined in the Elixhauser comorbidity index and all hospitalizations within two years prior to the first beta-blocker prescription. Comedications include all redeemed prescriptions within one year prior to the beta-blocker prescription. Kernel density estimations were used to assess the overlap in propensity and preference scores distributions for receiving carvedilol and thereby potential beta-blocker exchangeability. Totally, 10091 patients composed the study population; 2011 were exposed to carvedilol. The overlapping of propensity scores distributions was 57%. Accordingly, the exchangeability was not reached. Atrioventricular block (Odds Ratio, *OR* 8.20; 95% Confidence Interval, 95% *CI* 1.30–51.80), cerebrovascular thrombosis (OR 7.06; 95% CI 1.14–43.68), chronic kidney disease (OR 4.32; 95% CI 1.16–16.02), and acute heart failure (OR 1.97; 95% CI 1.28–3.03) hospitalizations were statistically significantly associated with carvedilol choice. Analogously, human insulin (OR 3.00; 95% CI 1.24–7.24), fondaparinux (OR 2.47; 95% CI 1.17–5.21) or strontium ranelate (OR 2.03; 95% CI 1.06–3.90) redeemed prescriptions. In conclusion, this study suggests the absence of beta-blockers exchangeability and a preferential choice of carvedilol in patients with heart failure, COPD and concurrent chronic kidney disease, atrioventricular block, cerebrovascular thrombosis, acute heart failure or redeeming human insulin, fondaparinux or strontium ranelate prescriptions. Therefore, it suggests that choice of prescribing carvedilol over metoprolol/bisoprolol/nebivolol is driven by differences in comorbidities and co-treatments.

## Introduction

Beta-blockade is a crucial pharmacological therapy to improve survival and to reduce morbidity in patients with heart failure and concurrent chronic obstructive pulmonary disease^1^. The benefits of beta-blockade in heart failure are due to their mitigation of the negative pathological consequences associated with adrenergic nervous system hyperactivity that gradually occurs in a chronically failing heart^2^. The sympathetic overdrive in heart failure is associated with an increased risk of arrhythmias, progressive dysfunction of the left ventricle, and myocardial insult, phenomena that have a negative impact on disease progression and prognosis^3^. In fact, adrenergic nervous system hyperactivity determines increased peroxidative and lipoperoxidative metabolism and demands of oxygen in the myocardium leading to the overproduction of reactive oxygen species that in turn determines local inflammation, apoptosis, and necrosis resulting in interstitial fibrosis and heart remodelling^4,5^. In Europe, four beta-blockers are currently indicated for heart failure, three beta-1 adrenoceptor selective, metoprolol, bisoprolol and nebivolol, and a non-selective, carvedilol. Metoprolol and bisoprolol are both second generation beta-blockers or rather they have high beta-1 adrenoceptor selectivity and they both lack intrinsic sympathomimetic activity. Bisoprolol has 20 times higher selectivity for the beta-1 adrenoceptor than metoprolol and it has a longer half-life (9–12 hours versus 3–7 hours) and bioavailability (90 versus 12%). Due to its high selectivity for the beta-1 adrenoceptor, bisoprolol has minimal effects on insulin sensitivity while, on the other hand, metoprolol has an additional membrane-stabilizing activity on the heart. Carvedilol and nebivolol are third generation beta-blockers with additional vasodilatation properties. Nebivolol is beta-1 adrenoceptor selective beta-blockers with a long half-life and bioavailability (12–96%) which, through a mechanism mediated by nitric oxide, is able to provide a reduction of the arteries tone that when targeted on the coronaries guarantees additional cardioprotective and anti-ischemic effects. Carvedilol is a non- beta-1 adrenoceptor selective beta-blockers with an intrinsic sympathomimetic activity, long half-life (7–10 hours) and additional vasodilatation properties mediated by the alpha-1 adrenoceptor blockade. It has anti-oxidant properties, it mitigates calcium overload by modulating ryanodine channels, and it ameliorates insulin sensitivity^6^. Despite a clinical and pharmacological rationale suggest the use of beta-blockers in heart failure, reluctance to prescribe beta-blockers in patients with the concurrent chronic obstructive pulmonary disease has been observed in multiple clinical settings. The leading factor for under-prescription of beta-blockers in this subpopulation is the concern of adverse respiratory effects mediated by beta-blockers through beta-2 adrenoceptors in the airway. The magnitude of the bronchoconstriction is strongly correlated with beta-1 adrenoceptor selectivity^7^. For this reason, current clinical guidelines suggest that for patients with heart failure and concurrent chronic obstructive pulmonary disease, metoprolol, bisoprolol or nebivolol should be preferred^8,9^. However, studies suggest a high proportion of carvedilol usage that is in opposition to above-mentioned recommendations^10^. Several adverse clinical consequences have been associated with carvedilol usage in patients with heart failure and concurrent chronic obstructive pulmonary disease such as a reduction in the lung function^11^ that may lead to subclinical effects on respiratory parameters^10,12,13^. Additionally, carvedilol may lead to a higher rate of unfavourable hemodynamic alterations leading to an increased risk of heart failure hospitalization, on both short and long-term following treatment commencement^10,12^. In virtue of the potential negative clinical consequences associated with carvedilol choice in patients with heart failure and chronic obstructive pulmonary disease naïve to beta-blockers, understanding key factors that may lead to a preferential choice of such prescriptions is crucial and, in this regard, the evidence is scarce. Our hypothesis is that concurrent chronic/acute comorbidities and comedications may have driven the choice of carvedilol over metoprolol/bisoprolol/nebivolol and this may have led to the absence of exchangeability of these beta-blockers in this subpopulation. To overcome this gap in knowledge, the current study aims to investigate carvedilol and metoprolol/bisoprolol/nebivolol exchangeability and the predictors of carvedilol choice in patients with heart failure and chronic obstructive pulmonary disease that were naïve to beta-blockers. The investigated predictors included chronic comorbidities defined in the Elixhauser comorbidity index^14^, all hospitalizations and redeemed prescriptions within two and one years prior to the first beta-blocker prescription, respectively.

## Methods

### Data sources

Health data sources of the Caserta Local Health Unit were used to identify the study population. These data sources cover approximately 1,000,000 citizens living in the catchment area of Caserta (Campania Region, Italy). For reimbursement purposes, the data sources store information on socio-demographic characteristics of residents, redeemed prescriptions, inpatient and outpatient hospitalizations and hospital contacts. Data contained in Caserta Local Health Unit administrative databases are pseudonymized to comply with current law on data protection and privacy.

### Study population

**Figure 1:**
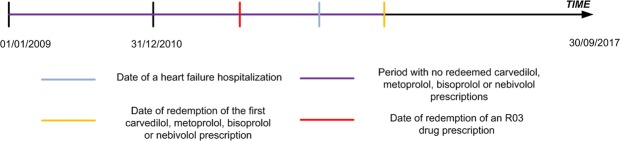
Example of the temporal sequence of events needed for patients to be included in the study population.

The study population included patients who (1) during the period December 31, 2010 to September 30, 2017 had a hospitalization/hospital contact for heart failure, (2) redeemed their first carvedilol, metoprolol, bisoprolol or nebivolol prescription after this hospitalization for heart failure (at least one year wash-out period for the beta-blocker prior to the heart failure hospitalization), and (3) redeemed drugs for obstructive airway diseases (Anatomical Therapeutic Chemical Classification, *ATC* R03) prior to the redemption of the first prescription of aforementioned beta-blockers (Fig. 1). In Italy, drugs for obstructive airway disease (ATC R03) that are reimbursed by the Italian Healthcare system are indicated for asthma or chronic obstructive pulmonary disease^12^. However, the use of beta-blockers in asthma is relatively contraindicated in the summaries of product characteristics and is not recommended in current clinical guidelines. Therefore, we believe that by using this approach we identified mostly patients with chronic obstructive pulmonary disease.

### Study outcome

The study outcome was the probability of redeeming a carvedilol as opposed to metoprolol/bisoprolol/nebivolol prescription.

### Potential predictors for carvedilol choice

Age, gender, year of inclusion in the study population and chronic comorbidities according to the Elixhauser Comorbidity Index were included as potential predictors^14^. Chronic comorbidities were identified by hospital discharge codes prior to the redemption of the first beta-blocker prescription. Other potential predictors included all primary diagnosis for hospital contact/hospitalization and redeemed prescriptions that occurred 2 and 1 year/s prior to the redemption of the first carvedilol, metoprolol, bisoprolol, or nebivolol prescription, respectively.

### Statistical analyses

A prevalence proportion (the prevalence of the event among patients exposed to carvedilol divided by the prevalence of the event among patients exposed to metoprolol, bisoprolol, or nebivolol) for all primary diagnosis for hospital contact/hospitalization and redeemed prescriptions that occurred 2 and 1 year/s prior to the redemption of the first carvedilol, metoprolol, bisoprolol, or nebivolol prescription was computed and the 500 event most prevalent among carvedilol users were selected. This is a crucial preliminary step to identify the most relevant variables for propensity score assessment in highly dimensional datasets. The 500 top-ranked potential predictors selected with the abovementioned method were used as independent variables in a logistic regression model to compute the propensity score for receiving carvedilol rather than metoprolol, bisoprolol, and nebivolol. The predicted probability of receiving carvedilol given the set of potential predictors was used to assess the exchangeability between treatments groups given their covariate distributions. The null hypothesis was the absence of clinical or demographic differences between recipients of carvedilol versus metoprolol, bisoprolol, or nebivolol or rather beta-blocker exchangeability (referred to as empirical ‘equipoise’ in Greenland *et al*.)^15^. According to Walker and colleagues, the predicted value from the above-mentioned logistic regression was used to compute a preference score as following: *ln* {*A*/(1 − *A*)} = *ln (B*/(1 − *B*) − *ln* (*C*/(1 − *C*)) where A is the preference score, B is the propensity score and C is the prevalence of exposure to carvedilol^16^. To assess beta-blocker exchangeability among beta-blockers, a Kernel density estimation was used to assess the overlap in propensity scores areas for carvedilol and metoprolol/bisoprolol/nebivolol users. Additionally, as suggested by Walker and colleagues, by using a Kernel density estimation, we tested if half of the distributions of the preference score was between 0.3 and 0.7 between carvedilol and metoprolol/bisoprolol/nebivolol users^16^. Baseline characteristics of the two cohorts were compared using the t-tests for continuous variables and χ^2^ test for categorical variables.

### Compliance with ethical standards

According to Italian National laws, ethical approval or informed consent for register-based studies is not required.

## Results

The study population’s demographic and clinical characteristics are provided in Table 1. In total, 10091 patients were included in the study population of which 2011 (19.9%) exposed to carvedilol. The mean age was 77.7 years (standard deviation of 11.8 years).

**Table 1:** Demographic and clinical characteristics of patients with heart failure and chronic obstructive pulmonary disease that received for the first time a prescription of carvedilol, metoprolol, bisoprolol or nebivolol and that were resident in the catchment area of Caserta Local Health Unit (Campania Region, Italy).

Variable	Metoprolol/Bisoprolol/Nebivolol (n = 8080)	Carvedilol (n = 2011)	Total (n = 10091)	p-value
Age	77.8 (11.7)	77.7 (11.9)	77.8 (11.8)	0.922
Sex (male)	4092 (50.7)	970 (48.2)	5062 (50.2)	0.079
Pulmonary embolism	77 (1.0)	16 (0.8)	93 (0.9)	0.595
Peripheral arterial disease	385 (4.8)	88 (4.4)	473 (4.7)	0.496
Liver disorders	400 (5.0)	88 (4.4)	488 (4.8)	0.309
Cancer	1248 (15.4)	306 (15.2)	1554 (15.4)	0.825
Chronic kidney disease	526 (6.5)	123 (6.1)	649 (6.4)	0.553
Arterial embolism	1640 (20.3)	373 (18.5)	2013 (19.9)	0.084
Diabetes mellitus type 2	1698 (21.0)	420 (20.9)	2118 (21.0)	0.922
Hypertension	2766 (34.2)	679 (33.8)	3445 (34.1)	0.711
Acute myocardial infarction	1214 (15.0)	290 (14.4)	1504 (14.9)	0.518
Atrial fibrillation	343 (4.2)	88 (4.4)	431 (4.3)	0.842
Ventricular fibrillation	423 (5.2)	104 (5.2)	527 (5.2)	0.953
Valvular Disease	291 (3.6)	70 (3.5)	361 (3.6)	0.846
Pulmonary Circulation Disorders	94 (1.2)	23 (1.1)	117 (1.2)	1.000
Depression	56 (0.7)	17 (0.8)	73 (0.7)	0.565
Alcohol Abuse	46 (0.6)	3 (0.1)	49 (0.5)	0.024
Obesity	240 (3.0)	85 (4.2)	325 (3.2)	0.005
Coagulopathy	30 (0.4)	6 (0.3)	36 (0.4)	0.778
Rheumatoid Arthritis	24 (0.3)	10 (0.5)	34 (0.3)	0.241
Solid Tumour without Metastasis	64 (0.8)	18 (0.9)	82 (0.8)	0.747
Lymphoma	15 (0.2)	4 (0.2)	19 (0.2)	1.000

### Beta-blocker exchangeability in patients with heart failure and concurrent chronic obstructive pulmonary disease

**Figure 2:**
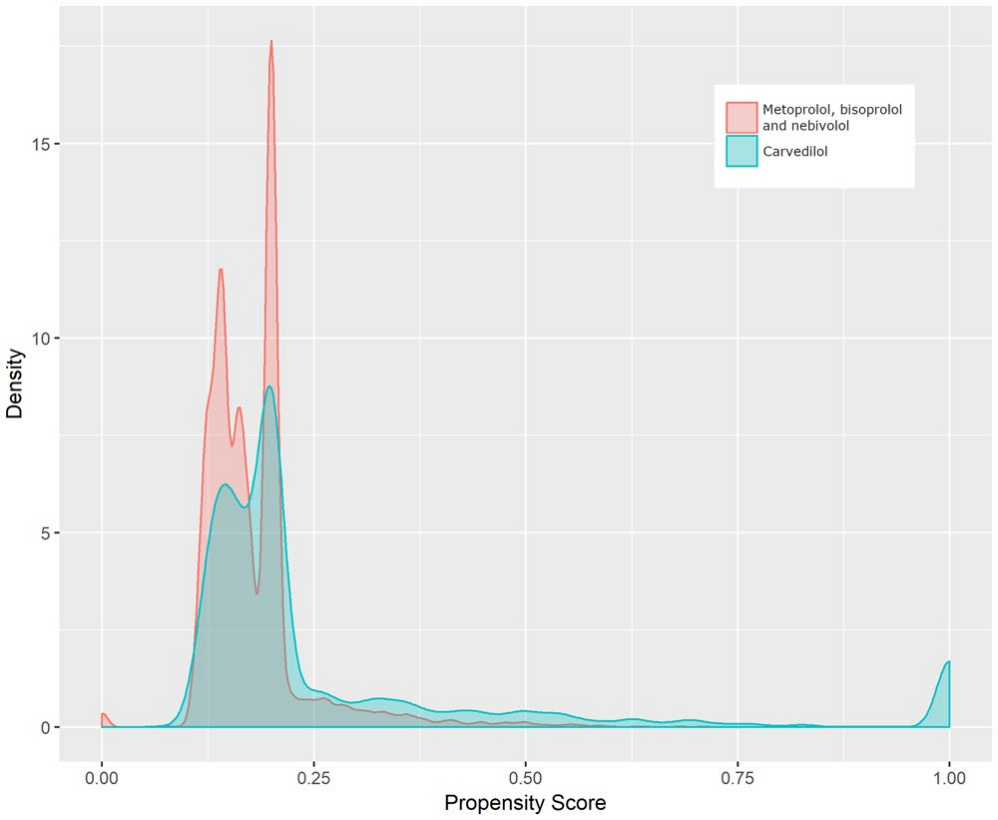
Kernel density estimations of propensity score among patients treated with carvedilol and metoprolol/bisoprolol/nebivolol having heart failure and chronic obstructive pulmonary disease located in the catchment area of Caserta Local Health Unit (Campania Region, Italy).

**Figure 3:**
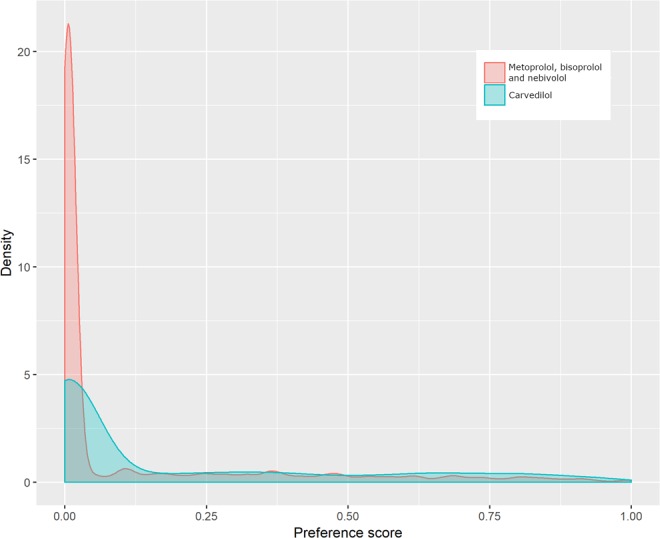
Kernel density estimations of preference score among patients treated with carvedilol and metoprolol/bisoprolol/nebivolol having heart failure and chronic obstructive pulmonary disease located in the catchment area of Caserta Local Health Unit (Campania Region, Italy).

The estimated overlapping area of two kernel density estimations of propensity scores among treated with carvedilol and metoprolol/bisoprolol/nebivolol was 57% (Fig. 2). The exchangeability among beta-blockers according to the preference score criteria was not reached (Fig. 3). Both results suggest the absence of exchangeability between treatment groups and therefore significant differences in comorbidities/co-treatments distributions among the 500 top-ranked potential predictors between patients receiving carvedilol over metoprolol/bisoprolol/nebivolol.

### Predictors of carvedilol choice

**Figure 4:**
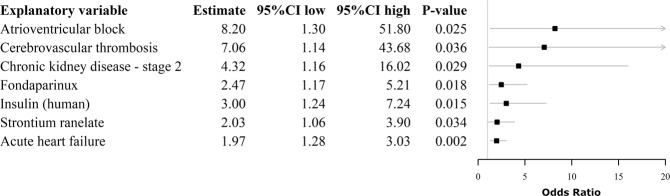
Predictors of carvedilol prescription over metoprolol/bisoprolol/nebivolol for patients with heart failure and chronic obstructive pulmonary disease located in the catchment area of Caserta Local Health Unit (Campania Region, Italy).

Odds ratios and 95% confidence intervals (95% CI) of statistically significant predictors for carvedilol prescription over metoprolol/bisoprolol/nebivolol prescriptions are provided in Fig. 4. Age, gender, and comorbidities according to the Elixhauser Comorbidity Index were not predictors of carvedilol choice. Among the 500 top-ranked potential predictors, atrioventricular block (OR 8.20; 95% CI 1.30–51.80), cerebrovascular thrombosis (OR 7.06; 95% CI 1.14–43.68), chronic kidney disease (OR 4.32; 95% CI 1.16–16.02), and acute heart failure (OR 1.97; 95% CI 1.28–3.03) hospitalizations/hospital contacts within two years prior to the first beta-blocker prescription were associated with carvedilol choice. Analogously, patients redeeming a prescription of human insulin, fondaparinux or strontium ranelate within one year prior to the beta-blocker prescription had significantly higher odds of receiving carvedilol over metoprolol/bisoprolol/nebivolol.

## Discussion

This study suggests that among patients with heart failure and chronic obstructive pulmonary disease, the choice of prescribing carvedilol over metoprolol/bisoprolol/nebivolol is driven by differences in comorbidities and co-treatments. Predictors of carvedilol choice were chronic comorbidities and co-treatments including chronic kidney disease, human insulin, fondaparinux, strontium ranelate or recent acute episode of acute heart failure, cerebrovascular thrombosis or atrioventricular block.

European clinical guidelines on chronic heart failure released during the study period do not state any preference among beta-blockers in the aforementioned clinical conditions and they do not provide recommendations in the clinical setting of multiple comorbidities. However, clinical evidence seems to support the choice of carvedilol over other beta-blockers in the clinical scenario listed above.

Carvedilol was found beneficial for patients with chronic kidney disease due to its ability to reduce cardiac output and renin release, as to its ability to reduce renovascular constriction through its alfa-1 and beta-1 receptor antagonism. Additional, carvedilol was useful due to its enhanced renovascular dilation through beta2-receptor antagonism^17^. These effects, along with to the ability of all beta-blockers in reducing sympathetic over-activity, were found associated with attenuation in the increase of albuminuria and hyperkalaemia, but also to an absence of an increase in serum creatinine, events typically observed in patients with chronic kidney disease. In addition, the aforementioned effects were found associated with high effectiveness in protecting from cardiovascular events in patients with chronic kidney disease^17–19^.

With regards to diabetes mellitus and in particular human insulin as a predictor for carvedilol choice, evidence suggests a favourable effect of carvedilol on glycaemic control in diabetic patients when compared to metoprolol. In particular, in the Glycaemic Effects in Diabetes Mellitus: Carvedilol-Metoprolol Comparison in Hypertensive (GEMINI) trial, patients treated with carvedilol had a lower probability of new onset diabetes mellitus, microalbuminuria (a surrogate marker for endothelial function) and an increased insulin sensitivity compared to patients treated with metoprolol. Additionally, a recent systematic review suggested even more beneficial effect of carvedilol in diabetes mellitus, including a reduced risk of new onset of atherogenic dyslipidaemia or weight gain among treated with carvedilol^20,21^.

Regarding the role of strontium ranelate as a predictor for carvedilol choice, it should be mentioned that our study population had a mean age of 77.8 years with a proportion of women of 49.8% and, therefore, at high risk of osteoporosis. Additionally, patients with chronic obstructive pulmonary disease have an increased risk of all-cause and site-specific fractures^22^. In Italy, during the study period, strontium ranelate was indicated for “*the treatment of severe osteoporosis in postmenopausal women and in adult men at high risk of fracture*”. Observational studies suggest an association between beta-blocker treatments, and in particular carvedilol, and reduced risk of bone fracture and an inherent protective effect of this drug class. On the other hand, pooled data from clinical trials that investigate the effect of carvedilol on congestive heart failure do not seem to support this hypothesis^23,24^. Mechanistic studies performed in pre-clinical models support the hypothetic involvement of β-adrenoceptor in the aforementioned phenomena by showing that beta-blocker treatment improves bone density and mass while β-adrenoceptor agonists led to the opposite effect. In particular, the effect seems mediated by the β2-adrenoceptor on osteoblasts and the sympathetic nervous system^25^. It should be mentioned that no formal potential drug-drug interactions were found between strontium ranelate and carvedilol or metoprolol/bisoprolol/nebivolol by using Micromedex^®^^26^. Therefore we excluded potential drug-drug interaction as a plausible explanation for carvedilol choice in these patients.

In relation to fondaparinux as a predictor of carvedilol prescription, we need to mention that during the study period, fondaparinux was indicated for the prevention of venous thromboembolism in multiple clinical scenarios. We believe that a plausible explanation for the higher proportion of fondaparinux use in patients treated with carvedilol may be due to the fear of potential drug-drug interactions. In particular, clinical guidelines advocate the use of fondaparinux for venous thromboembolism prophylaxis in patients with a history of heparin-induced thrombocytopenia^27^. Alternatives to fondaparinux in the aforementioned clinical scenario are the direct acting oral anticoagulants^27^. However, while carvedilol has no potential drug-drug interactions with fondaparinux^26^, it has with direct acting oral anticoagulants, and in particular dabigatran and edoxaban. Given carvedilol’s ability to inhibit the glycoprotein-P, it may induce an increased plasma concentration of the aforementioned direct acting oral anticoagulants with a consequent increased risk of bleeding^26^. Therefore, we speculate that the occurrence of these clinical scenarios may have led the choice of carvedilol in patients exposed to fondaparinux.

In relation to acute heart failure, previous studies have found different effects on the cardiovascular system among beta-blockers with different β-1 adrenoceptor selectivity in patients that experienced acute heart failure and that were exposed to inotropic agents (i.e. dobutamine). In this subpopulation, metoprolol did not modify the cardiac index, heart rate, and systemic vascular resistance providing only a modest effect on the pulmonary artery and systemic arterial pressures within the first 12 months of treatment. In contrast, substantial differences were observed for carvedilol. The aforementioned effects were attributed to the blockade of β-2-receptors in the vascular system and to the other pharmacological properties of carvedilol^28,29^.

It should be mentioned that clinical evidence exists on the beneficial effect of beta-blockers on the risk of early death in patients with ischemic stroke^30^. In pre-clinical and *in-vitro* models, authors found neuroprotective effects of beta-blockers with additional vasodilatation properties, like carvedilol and nebivolol. Notably, for carvedilol, a study suggests that these effects may be mediated also by the β2 adrenal receptors antagonism and cyclooxygenase 2 isozyme/prostacyclin pathways^31,32^. Additionally, clinical evidence seems to support the hypothesis of a reduced risk of mortality due to stroke in patients exposed to carvedilol versus those exposed to metoprolol^33^.

Regarding the choice of carvedilol in patients with a recent episode of atrioventricular block, it should be highlighted that clinically relevant differences were observed among beta-blockers indicated for heart failure on the heart rate. In particular, among aforementioned beta-blockers, carvedilol provided a weaker effect on heart rate at rest when compared to other beta-blockers especially in patients with a low sympathetic tone which may be beneficial in patients with a recent episode of atrioventricular block. It was hypothesized that this effect might be caused by the increased sympathetic drive consequent to the peripheral vasodilation resulting from the accessories vasodilatation properties of carvedilol^34,35^.

### Strengths and limitations

A major limitation of this study is the lack of information on the indication of use for redeemed prescriptions. For drugs having ATC code R03, it may have led to a misclassification of the exposure. In fact, these drugs are mainly indicated for both asthma and chronic obstructive pulmonary diseases. Therefore, we may have classified patients with asthma as having chronic obstructive pulmonary disease. We believe that the magnitude of this type of misclassification is very low because Caserta Local Health unit databases contain information on drugs reimbursed by the national health system, and most of the reimbursed drugs in this ATC group are indicated for chronic obstructive pulmonary disease as shown elsewhere^36^. Additionally, beta-blockers are relatively contraindicated in asthma.

The second major limitation of this study is the lack of information on over-the-counter drugs/drugs not requiring a medical prescription. In particular, we may have not detected predictors for carvedilol choice among acute comorbidities. In the Italian healthcare system, over-the-counter drugs/drugs not requiring a medical prescription are generally not indicated for chronic and/or invalidating diseases with a high burden for the patient and they are usually used as symptomatic treatments^37^. Another limitation of this study is the potential misclassification of chronic comorbidities identified through hospitalizations/hospital contacts. In fact, we lack information for those patients hospitalized and/or with a hospital contact outside the catchment area of Caserta local health unit. However, we believe that the magnitude of this bias is low because these patients will receive in most of the cases a pharmacological treatment that is tracked in Caserta Local health unit databases.

The third major limitation of this study is the lack of information on the ethnicity/race of the study population for which, recent studies found an association with a differential response to beta-blockers treatment^38,39^. However, we believe that this limitation may have introduced a negligible bias. In fact, during the study period, the number of immigrants in the catchment area of Caserta was between 3000–4000, or rather 0.3–0.4% of the study population and, mostly with an age lower than 60 years and, therefore, with a lower probability of having heart failure^40^.

Regarding the lack of information on the cytochrome P450 2D6 polymorphisms in the study population, despite carvedilol is extensively metabolized by this enzyme^41,42^, to date, in Europe, none of the summaries of product characteristics of medicinal products containing carvedilol include polymorphisms of the gene codifying this enzyme as a “*contraindications*”/“*special warnings and precautions for use*” for its prescription. Therefore, we believe that even in the setting of poor, rapid or ultra-rapid metabolizers, it should not have influenced the prescription choice.

Finally, as for all observational studies, we cannot exclude the presence of unmeasured confounders that may have introduced biases in our analysis. Strengths include the use of the entire population of patients enlisted in Caserta local health unit databases reducing the risk of selection bias and the possibility of providing results based on real-world data from routine clinical practice.

## Conclusion

In conclusion, this study showed that no beta-blocker exchangeability was found among carvedilol and metoprolol/bisoprolol/nebivolol in patients with heart failure and chronic obstructive pulmonary disease. In this subpopulation, concurrent chronic (i.e. chronic kidney disease) and acute comorbidities (i.e. acute heart failure, cerebrovascular thrombosis or atrioventricular block) or concurrent pharmacological treatments (i.e. fondaparinux, human insulin and strontium ranelate) may drive the choice of prescribing carvedilol over metoprolol/bisoprolol/nebivolol.

## Data Availability

The datasets generated during and/or analyzed during the current study are not publicly available due to legal reservations but are available from the corresponding author on reasonable request.
